# On the origin of TSAR: morphology, diversity and phylogeny of Telonemia

**DOI:** 10.1098/rsob.210325

**Published:** 2022-03-16

**Authors:** Denis V. Tikhonenkov, Mahwash Jamy, Anastasia S. Borodina, Artem O. Belyaev, Dmitry G. Zagumyonnyi, Kristina I. Prokina, Alexander P. Mylnikov, Fabien Burki, Sergey A. Karpov

**Affiliations:** ^1^ Papanin Institute for Biology of Inland Waters, Russian Academy of Sciences, Borok, Russia; ^2^ Department of Organismal Biology, Program in Systematic Biology, Uppsala University, Uppsala, Sweden; ^3^ Science for Life Laboratory, Uppsala University, Uppsala, Sweden; ^4^ Department of Zoology and Parasitology, Voronezh State University, Voronezh, Russia; ^5^ Department of Zoology and Ecology, Penza State University, Penza, Russia; ^6^ Ecologie Systématique Evolution, CNRS, Université Paris-Saclay, AgroParisTech, Orsay, France; ^7^ Zoological Institute, Russian Academy of Sciences, Saint Petersburg, Russia; ^8^ Department of Invertebrate Zoology, Faculty of Biology, Saint Petersburg State University, Saint Petersburg, Russia

**Keywords:** protists, telonemids, 18S rDNA, microbial diversity, ultrastructure, evolution

## Abstract

Telonemia is a poorly known major phylum of flagellated eukaryotes with a unique combination of morphological traits. Phylogenomics recently revealed the phylogenetic position of telonemids as sister to SAR, one of the largest groups of eukaryotes, comprising Stramenopiles, Alveolata and Rhizaria. Due to this key evolutionary position, investigations of telonemids are of critical importance for elucidating the origin and diversification of an astounding diversity of eukaryotic forms and life strategies. To date, however, only two species have been morphologically characterized from Telonemia, which do not represent this genetically very diverse group. In this study, we established cultures for six new telonemid strains, including the description of five new species and a new genus. We used these cultures to update the phylogeny of Telonemia and provide a detailed morphological and ultrastructural investigation. Our data elucidate the origin of TSAR from flagellates with complex morphology and reconstruction of the ancestral structure of stramenopiles, alveolates and rhizarians, and their main synapomorphic characters. Since telonemids are a common component of aquatic environments, the features of their feeding, behaviour and ecological preferences observed in clonal cultures and the results of global metabarcoding analysis contribute to a deeper understanding of organization of microbial food webs.

## Graphical abstract

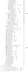

## 1. Introduction

The eukaryote tree of life has been extensively rearranged by phylogenomics and the addition of numerous new ‘kingdom-level’ lineages of heterotrophic protists [1]. The supergroup SAR (containing Stramenopiles, Alveolata, Rhizaria) was established in 2007, at the dawn of the phylogenomic era [2–4]. SAR may comprise up to half of all eukaryote species diversity [5,6], including a wide array of morphologies and ecology such as heterokont algae (e.g. brown, diatom and golden algae), dinoflagellates, ciliates, foraminiferans, radiolarians, oomycete and apicomplexan parasites, and many other important free-living and parasitic microorganisms. Until recently, little was known about the closest relatives (sister group) of SAR, which impeded inferences about the evolutionary origins of this supergroup and its colossal diversity. However, a phylogenomic analysis of the mysterious predatory flagellates Telonemia showed that this lineage is the sister of SAR, which were proposed to form the mega-assemblage TSAR (Telonemia + SAR) [7]. Telonemia thus represent an opportunity to study the origin and the evolution of morphological synapomorphies and fundamental cellular innovations (e.g. cytoskeleton) of the SAR supergroup, and more generally to contribute to our understanding of diversification of eukaryotes and their life strategies.

Despite having only recently found a place on the eukaryotic tree [7], telonemids have been long known, with the first species—*Telonema subtile* (syn. *T. subtilis*)—described over a century ago from crude cultures of *Ulva* and of red algae from Roscoff and Naples [8]. Following this initial description, only one other species has been described—*T. antarcticum*—from the surface marine waters of the inner Oslo fjord [9], which was later renamed *Lateronema antarctica* due to the morphological and 18S rDNA differences compared with *Telonema* [10]. In addition to these two named species, about 100 undescribed lineages of Telonemia have been identified in marine plankton and freshwater environments [11]. The phylogenetic analysis of these environmental lineages showed 20 statistically supported subclades of telonemids composed of phylotypes from different geographic regions [11]. Being widely distributed and sometimes abundant, telonemids probably play important ecological functions in water ecosystems [9,12–15]. The huge diversity of telonemid phylotypes is currently uncharacterized morphologically.

Available data on the morphology of the two known species of telonemids indicate that they are phagotrophic biflagellate protists of pyriform shape with flagella emerging on opposite sides of a short protruding antapical rostrum or proboscis [16]. Telonemids feed on a wide range of bacteria and pico- to nano-sized phytoplankton [11]. They possess tubulocristate mitochondria and a unique, highly complex multi-layered cytoskeleton composed of layers of microtubuli and microfilaments [9,17]. Strikingly, Telonemia exhibit a unique combination of cellular structures that have only been found separately in different groups of SAR as well as some other eukaryotic lineages such as kathablepharids, heliozoans and excavates [16,17]. For example, *Lateronema* possesses tripartite mastigonemes and alveoli-like structures [9] that might be homologous to those of stramenopiles and alveolates, respectively. Thus, telonemids possess peculiar external morphology and very intricate ultrastructural organization that are some of the most complex among eukaryotes.

In this study, we have isolated six new strains of telonemids from different and geographically distant marine and freshwater habitats. We present a detailed analysis of the ultrastructure and morphology of these new isolates, including the description of new genus and five species, and clarify the phylogenetic relationships within telonemids based on the 18S ribosomal RNA gene (18S rRNA). We also analysed the geographical distribution and abundance of telonemids on the base of environmental sequences and their growth dynamics in relation to the temperature and salinity. Due to its key evolutionary position, the study of telonemids can help in elucidating the origin of TSAR and reconstruction of the ancestral structure of stramenopiles, alveolates and rhizarians, as well as in clarification of evolutionary speciation and ecological distribution of descendants of the SAR ancestor.

## 2. Material and methods

### 2.1. Clone isolation and culture maintenance

Clonal cultures of telonemids *Arpakorses versatilis* gen. et sp. nov. (clone P-1), *Arpakorses idiomastiga* gen. et sp. nov. (clone P-2) and *Telonema subtile* (clone Tel-1) were isolated from Arctic waters of the Kara Sea as described before [7]. Freshwater clone Tel-2 (*Telonema papanine* sp. nov.) was isolated from the sample of moss and sand in the glacier melting zone (70 m from the water edge) on Champ Island, Franz Josef Land archipelago, Arctic Ocean (80°37'46.8″ N, 56°53'45.5″ E) on 26 July 2019. Clone Tel-3 (*Telonema tenere* sp. nov.) was obtained from the sand of the marine littoral zone of the White Sea, 0.5 m from the shoreline (66°29'58.787″ N, 35°9'42.206″ E) on 11 August 2020. Clone Tel-4 (*Telonema rivulare* sp. nov.) was isolated from the bottom detritus with sand of Sakhray River, Republic of Adygea, Russia (44°10'26.7″ N, 40°17'58.8″ E) on 3 September 2020 (water temperature 25.5°С, electric conductivity 289 µS cm^−1^, рН 8.5).

The samples were enriched with a suspension of *Pseudomonas fluorescens* Migula, 1895 bacteria and examined on the third, sixth and ninth day of incubation in accordance with methods described previously [18]. Following isolation by glass micropipette, clones of telonemids were propagated on the bodonids. *Procryptobia sorokini* (Zhukov, 1975), Frolov *et al.* 2001, feeding on *Pseudomonas fluorescens* bacteria and grown in 20‰ artificial marine medium (RS-R11040, Red Sea), was used as prey for marine telonemids (clones P-1, P-2, Tel-1, Tel-3). *Parabodo caudatus* (Dujardin, 1841), Moreira *et al*., 2004, feeding on *Pseudomonas fluorescens* bacteria and grown in mineral water (Aqua Minerale, PepsiCo), was used as prey for freshwater clones Tel-2 and Tel-4. Telonemid cultures are currently being stored in a collection of live protozoan cultures at the Papanin Institute for Biology of Inland Waters, Russian Academy of Sciences.

### 2.2. Microscopy

Light microscopy observations were made using an AxioScope A.1 (Carl Zeiss, Germany) equipped with a DIC and phase-contrast water immersion objectives (63×) and an AVT HORN MC-1009/S analogue video camera and MC-12 digital camera (Lomo-Microsystems, Russia).

For investigation of cell ultrastructure in transmission electron microscope (TEM), cells were centrifuged for 20 min at 5000*g*; 0.5 ml of 4% glutaraldehyde (in 0.1 M cacodylate buffer) was added to the 0.5 ml of resuspended cells and kept at +4°С for 2 h. The pellet of fixed cells was embedded in 1% agarose and rinsed twice by 10 min with cold (+4°С) 0.1 M cacodylate buffer. After fixation in cold (+4°С) 1% osmium tetroxide in 0.1 M cacodylate buffer for 1 h, a pellet was rinsed with 0.1 M cacodylate buffer for 10 min. After dehydration in alcohol series (30, 50, 70 and 96%) and propylene oxide a pellet was embedded in Spurr resin (EM 0300 Sigma-Aldrich). Ultrathin sections (60 nm) were prepared with an Leica EM UC6 ultramicrotome (Leica Microsystems, Germany) and observed using the JEM-1011 TEM (JEOL, Japan).

Whole-mount cells of telonemids were also examined in TEM. Drops of cell cultures were placed on formvar-coated TEM grids and fixed in vapours of 2% osmium tetroxide for 10 min. After rinsing with distilled water, the cells were stained in 1% uranyl acetate (С_4_H_6_O_6_U) for 10–20 min and rinsed with distilled water again. Whole-mount preparations were observed by using the JEM-1011 and JEM-100C TEMs (JEOL, Japan).

For scanning electron microscopy (SEM), cells were harvested by centrifugation (7000*g*); 0.5 ml of 2.5% glutaraldehyde (in 0.1 M cacodylate buffer) was added to the 0.5 ml of resuspended cells and kept at room temperature for 20 min. Fixed cells were gently drawn onto a polycarbonate filter (pores 1 µm). Then, cells were rinsed with 0.05 M cacodylate buffer for 10 min and taken through a graded ethanol dehydration (30, 50, 70 and 96%) followed by propylene oxide and finally put overnight into a hexamethyldisiloxane. For the cells of the Tel-2 clone, 4% glutaraldehyde was used for fixation, as well as acetone instead of propylene oxide for dehydration. Dry filters were mounted on aluminium stubs, coated with gold and observed with JSM-6510LV (JEOL, Japan).

### 2.3. Laboratory experiments

Laboratory experiments were performed to determine the effects of temperature and salinity on the growth dynamics of *Arpakorses versatilis* gen. et sp. nov. and *Telonema subtile*. A certain number (ind. ml^−1^) of telonemids was added to a certain number of prey cells (*Procryptobia sorokini*). In the experiment with the temperature factor, 4.5 ml of such diprotist cultures were incubated in Petri dishes (6 cm in diameter) in thermostats at the following temperatures: 0°C, 5°C, 10°C, 15°C, 20°C, 25°C and 30°C. In the experiment with a factor of salinity, 4.5 ml of diprotist cultures were incubated at 10°C in a mineral water, Aqua Minerale, PepsiCo (0‰) and artificial sea water medium with salinities 5‰, 10‰, 20‰, 30‰, 50‰ and 100‰. The experiments were carried out for 20 days in three replicates. Live cells count was made using inverted AxioObserver 5 (Carl Zeiss, Germany) microscope.

### 2.4. 18S rRNA gene sequencing

Cells were harvested from Petri dishes following peak abundance after eating most of the prey. Cells were collected by centrifugation (1000*g*, room temperature) onto the 0.8 µm membrane of a Vivaclear mini-column (Sartorium Stedim Biotech Gmng, cat. no. VK01P042). Genomic DNA was isolated using the MasterPure Complete DNA and RNA Purification Kit (Epicentre, cat. no. MC85200). The 18S rRNA genes were amplified using the EconoTaq PLUS GREEN 2X Master Mix (Lucigen, cat. no. 30033-1) and universal eukaryotic primers PF1-FAD4 (for clones P-1, Tel-1, Tel-2), EukA-EukB (P-2) and 25F-1801R (Tel-3, Tel-4) [19–21]. Amplified DNA fragments were purified with QIAquick PCR Purification Kit (Qiagen, cat. no. 433160764). PCR products were subsequently cloned (Tel-2, Tel-3, Tel-4) using Quick-TA kit (Cat. no. TAK02, Evrogen) or sequenced directly (Tel-1, P-1, P-2) using Sanger dideoxy sequencing. Two additional internal primers 18SintF (5′-GGTAATTCCAGCTCCAATAGCGTA-3′) and 18SintR (5′-GTTTCAGCCTTGCGACCATACT-3′) were used for sequencing reaction. The resulting sequences were assembled from four overlapping reads using the Geneious R6 6.0.6 program.

### 2.5. Telonemia phylogenetic analyses

To assess the diversity of Telonemia, in addition to the sequences generated in this work, we collected all Telonemia sequences available in GenBank, following the EukRef pipeline (https://pr2-database.org/eukref/pipeline_overview/) [22]. Briefly, we used the Telonemia dataset from Bråte *et al.* [11] as a starting sequence set and BLASTed against GenBank in a loop until no new sequences were found, using a similarity threshold of 90%. We detected and removed chimeras from the retrieved sequences using UCHIME and the SILVA database as reference [23,24]. This resulted in 519 sequences that were at least 500 bp long. These sequences were clustered at 99% similarity to reduce redundancy, resulting in 144 sequences which were then pooled together with the sequences generated in this study as well as outgroup sequences. An alignment was generated using mafft-linsi and trimmed with trimal (−gt 0.3, −st 0.001), resulting in a final dataset comprising 159 taxa and 1698 aligned sites [25,26]. A maximum-likelihood phylogeny was inferred with IQTREE v. 1.6.3 along with 1000 ultrafast bootstrap replicates under the GTR + Gamma mode [27]. For Bayesian analyses, the Covarion and GTR + I models were used, and two independent runs of four chains were set up in MrBayes v. 3.2.7a for 20 million generations, sampling every 2000 generations [28]. For each run, 2500 trees were discarded as burn-in.

### 2.6. Assessing environmental diversity

To investigate the global distribution of telonemids in marine waters and to determine which clades were the most abundant and most diverse, we used the Tara Oceans and Malaspina Expedition metabarcoding dataset corresponding to the V9 and V4 regions of the 18S rDNA gene, respectively [29,30]. We obtained the Tara Oceans OTU dataset from Richter *et al.* [31]. This contained 739 million metabarcodes from 113 stations across the global surface ocean from which we retrieved 327 OTUs that were annotated as telonemids. We extracted 76 sequence variants (ASVs) from the Malaspina dataset. Global distribution plots were generated in R using ggplot [32].

To phylogenetically place the metabarcodes, we first generated a reference phylogeny; a subset alignment containing 74 sequences that spanned the V9 region was generated from the full alignment (see the previous section), and a maximum-likelihood phylogeny was inferred with RAxML-NG under the GTR + Gamma mode [33]. The V4 and V9 sequences were aligned against the reference alignment with PaPaRa [34] and subsequently placed on the reference phylogeny using the Evolutionary Placement Algorithm as implemented in RAxML v. 8.2.10 [35]. Placements were then assessed and visualized with the tool Gappa [36].

## 3. Results and discussion

### 3.1. Phylogeny, diversity and distribution of telonemids

**Figure 1.**
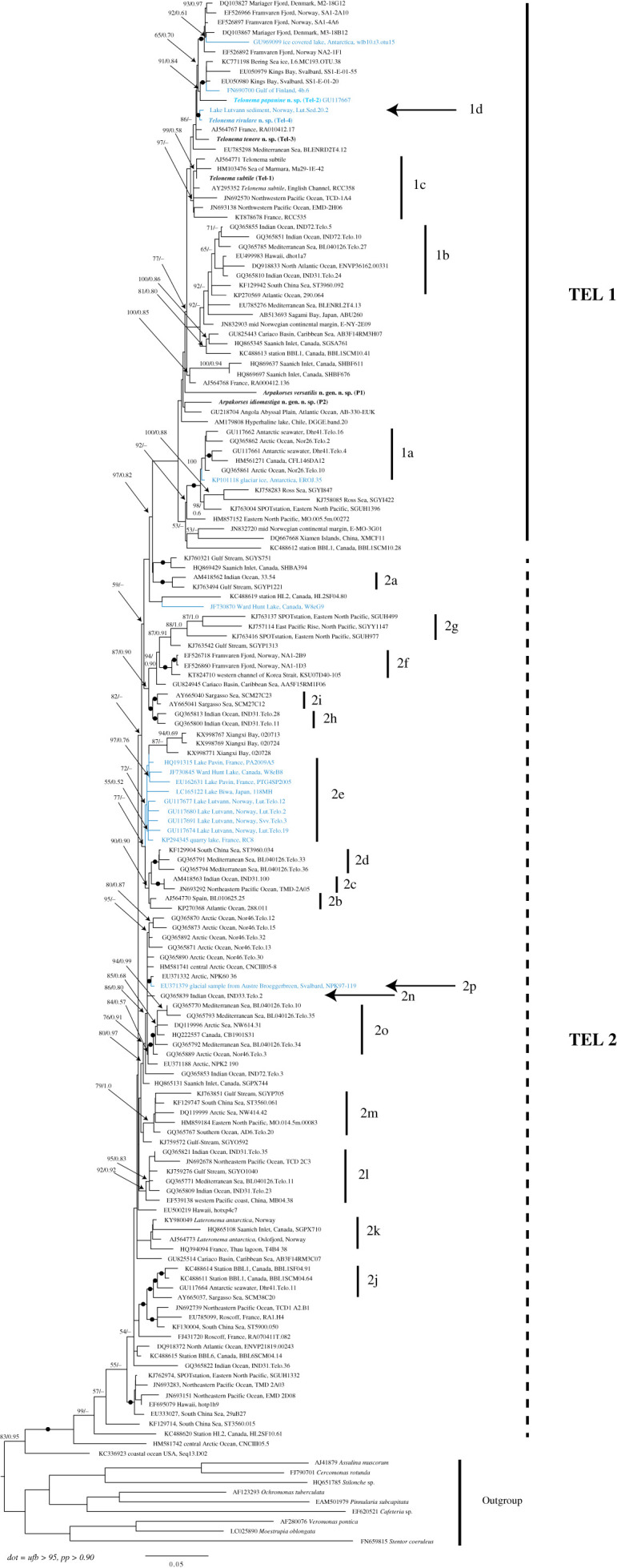
The phylogeny of telonemids. Maximum-likelihood and Bayesian phylogenies of 18S rRNA sequences. Numbers at the nodes represent Ultrafast bootstrap (IQTree) and Bayesian support values, respectively. Names of the groups refer to clades that were recognized in Bråte *et al.* [11]. Only values above 50/0.50 are shown and black dots indicate strong statistical support (≥95/0.90). Taxa labels in blue indicate freshwater sequences.

The phylogeny of telonemids has been characterized by a relatively large diversity of sequences divided into two main groups, named TEL 1 and 2 [11]. Our analysis of 144 18S rRNA sequences of Telonemia retrieved from GenBank using the EukRef pipeline [22] and the six new sequences obtained in this study did not recover this division, but instead showed TEL 1 originating from within a paraphyletic TEL 2 (figure 1). Consistent with Bråte *et al.* [11], however, we recovered the 20 proposed subgroups (1a–1d and 2a–2p; but not always with significant support), each possibly representing several species. The TEL 1 clade includes 50 environmental sequences, *Telonema subtile*, and all new six isolated strains as part of this study. One of them corresponds to *T. subtile,* while three strains represent new species of *Telonema* (*T. papanine* sp. nov., *T. rivulare* sp. nov. and *T. tenere* sp. nov.). We also describe two strains (P-1 and P-2) less related to *Telonema,* which we propose from the new genus *Arpakorses* gen. nov. within Telonemia with two novel species (*A. versatilis* gen. et sp. nov. and *A. idiomastiga* gen. et sp. nov.).

Freshwater *T. papanine* sp. nov. from Franz Josef Land is closely related to a diversity of environmental sequences from the sea ice of Bering and Baltic Sea and marine sediments of Svalbard. *Telonema rivulare* sp. nov. from the riverine bottom detritus of Adygea (southern Russia) is related to the environmental sequence from Lutvann Lake sediments in Norway. 18S rRNA sequence of marine benthic *T. tenere* sp. nov. from the White Sea is most similar to the environmental sequence from France picoplankton. *T. subtile* strain Tel-1 isolated from the Arctic Kara Sea is closely related to the *T. subtile* from the English Channel and environmental sequence from the Sea of Marmara. *Arpakorses idiomastiga* gen. et sp. nov. from the Arctic Kara Sea was related to an environmental sequence from deep-sea (5655 m) abyssal of the southeast Atlantic.

While telonemids were initially found to be exclusively marine organisms, targeted environmental sequencing revealed the presence of freshwater lineages as well [11]. Our analysis identified 15 freshwater OTUs across the telonemid diversity (highlighted in blue in figure 1) from water bodies in different regions (Antarctica, Norway, Finland, Canada, Japan, France). Given that freshwater environments remain less studied than marine habitats, it is likely that new lineages will be discovered as more freshwater samples are analysed. The new species *T. papanine* sp. nov. and *T. rivulare* sp. nov. are the first morphologically identified representatives of telonemids inhabiting freshwater. No biogeographic patterns were revealed in the grouping of sequences into phylogenetic clades. Generally, they do not correspond to geographical or climatic regions. However, it is noteworthy that the majority of known environmental sequences and most of the clones isolated here originate from cold-water sites of both hemispheres, although environmental sequences from tropical regions (Hawaii, the South China Sea, Caribbean Sea, etc.) were also revealed.

In order to test the relationship of telonemids to temperature and salinity, we carried out laboratory experiments with the two genera available in clonal cultures and show that the optimal growth temperature for *Arpakorses versatilis* gen. et sp. nov. and *T. subtile* is 10°C (electronic supplementary material, figure S1*a*,*b*). Both did not survive at temperatures above 25°C but tolerated cold marine water (0°C). The optimal salinity values for growth of *A. versatilis* gen. et sp. nov. and *T. subtile* are 20‰ and 30‰, respectively, and they did not survive in freshwater and brackish water (below 5‰ for *A. versatilis* gen. et sp. nov. and 10‰ for *T. subtile*), nor did they tolerate hypersaline conditions of 50‰ and above (electronic supplementary material, figure S1*c*,*d*).

**Figure 2.**
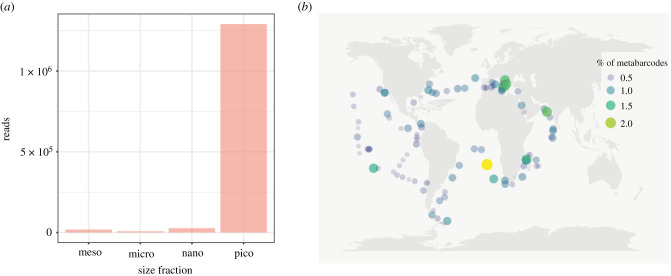
Distribution of telonemids in the World Ocean. Metabarcoding data from 113 stations collected during the Tara Oceans expedition and corresponding to the V9 fragment of the 18S rRNA gene. (*a*) Telonemids were found to be enriched in the pico size fraction (0.8–5 µm), which is consistent with their cell sizes. (*b*) Percentage of telonemid metabarcodes recovered in the pico size fraction.

To gain a better understanding of the distribution of telonemids in the oceans, we identified telonemid sequences in planktonic metabarcoding data of different size fractions in the Tara Oceans database. This analysis revealed that telonemids are most common in the picoplanktonic (0.8–5 µm) size fraction (figure 2*a*), and that this group is present in almost all oceanic regions, with predominance in the southeast Atlantic (figure 2*b*). The relative abundance of telonemids in the picoplankton fraction is variable between regions (figure 2*b*); the largest share is in the southeastern Atlantic (2% of the total metabarcodes of all unicellular eukaryotes), followed by the Mediterranean Sea, the Mozambique Strait and the Arabian Sea (up to 1.5%). In general, the results of the analysis of environmental sequences show that telonemids inhabit both marine and freshwaters of various geographical zones.

**Figure 3.**
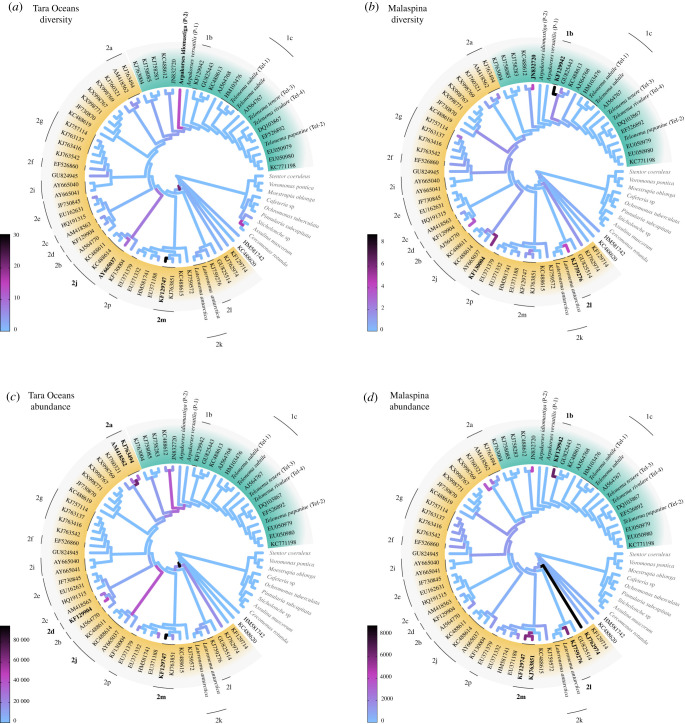
Phylogenetic placement of Tara Oceans and Malaspina expedition telonemid OTUs (*a,b*) and metabarcodes (*c,d*) on the Telonemia reference tree. The branch colours represent the distribution of OTU/metabarcode placement with darker branches indicating (*a,b*) that more OTUs were placed on them or (*c,d*) that those particular lineages are more abundant in the global ocean. TEL 1 is depicted in green, and TEL 2 in yellow. Specific clades in each TEL group are indicated with black lines.

Phylogenetic placement of the Tara Oceans and Malaspina Expedition OTUs on the Telonemia reference tree (figure 3*a–d*) revealed different trends in the global marine diversity and abundance of telonemids. Most of the Tara Oceans diversity is represented by clades 2m, 2j [11] and, interestingly, the newly described species *A. idiomastiga* gen. et sp. nov. (figure 3*a*), while the Malaspina Expedition diversity (figure 3*b*) is best represented by clades 1b, 2l and environmental sequences from the South China Sea and Norwegian Sea (KF130004, JN832720). In addition, we found that around 20 OTUs from the Tara Oceans were placed on internal branches with high likelihood weight ratios, indicating potential deep branching novel diversity (figure 3*a*). Furthermore, a few V9 OTUs were phylogenetically placed in the SAR clade, which may indicate sequence artefacts or the existence of species closely related to telonemids and SAR. Both global datasets indicate that clades 2m and 2a contain abundant OTUs (figure 3*c,d*). However, clade 2d was only found to be abundant in the Tara Oceans dataset while 2l, 1b and deep branching sequence KJ762974 were highly abundant exclusively in the Malaspina dataset.

### 3.2. External morphology of the six new telonemid isolates

#### 3.2.1. *Arpakorses versatilis* gen. et sp. nov.

**Figure 4.**
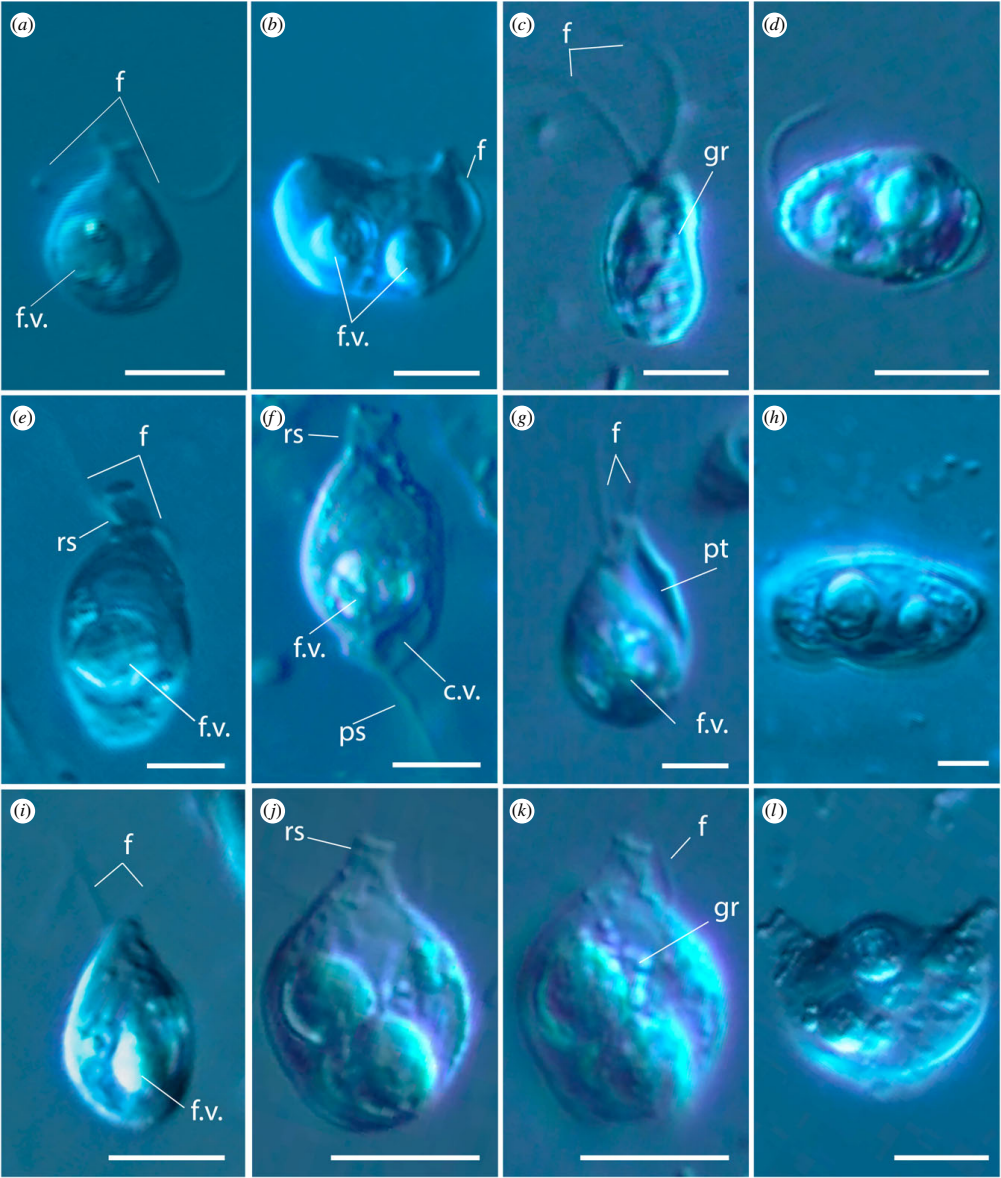
External morphology of telonemids, differential interference contrast microscopy. *Arpakorses versatilis*, attached cell (*a*) and cell division (*b*). *Arpakorses idiomastiga*, typical cell (*c*) and cell division (*d*). *Telonema papanine*, typical cell (*e*) and cell with pseudopodium (*f*). *Telonema tenere*, typical cell (*g*) and cell division (*h*). *Telonema rivulare*, typical cells (*i*–*k*) and cell division (*l*). Scale bars: 5 µm. Abbreviations: c.v.—contractile vacuole; f—flagella; f.v.—food vacuole; gr—granules; pt—pit; ps—pseudopodium; rs—rostrum.

**Figure 5.**
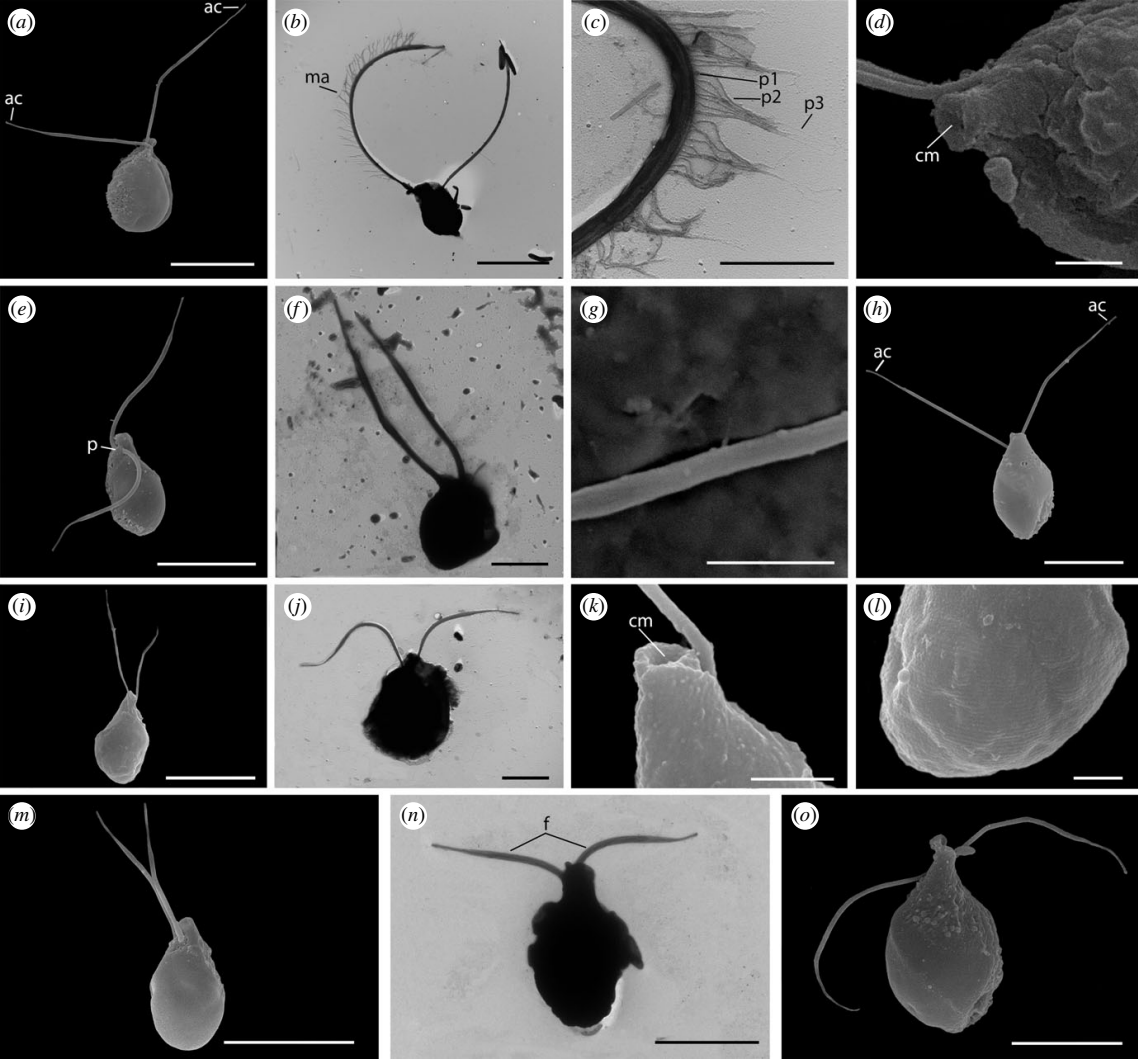
External morphology of telonemids, SEM. (*a*–*d*) *Arpakorses versatilis*, (*e*–*g*) *Arpakorses idiomastiga*, (*h*) *Telonema subtile*, (*i*–*l*) *Telonema papanine*, (*m*–*n*) *Telonema tenere*, (*o*) *Telonema rivulare*. Scale bars: (*a*,*b*,*e*,*f*,*h*,*i*,*j,m*,*n,o*)—5 µm; (*c*,*d*,*g*,*k*,*l*)—1 µm. Abbreviations: ac—acroneme; cm—cytostome; f—flagella; ma—mastigoneme; p—protrusion; p1,p2,p3—part 1,2,3 of mastigoneme.

**Figure 6.**
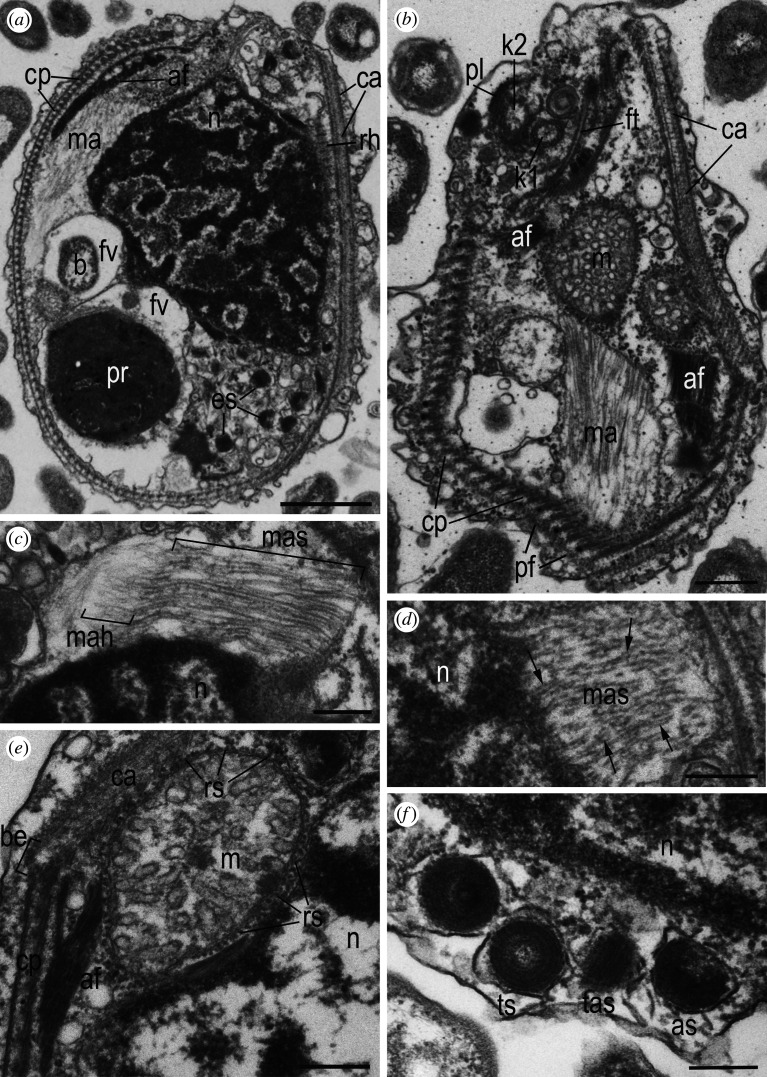
General cell organization of (*a*,*b*,*d*,*e*) *Arpakorses versatilis* and (*c*,*f*) *Arpakorses idiomastiga*. (*a*) General view of the cell at LS. (*b*) Cell structures at its anterior end. (*c*) Mature mastigonemes in the dilation of perinuclear space at LS and transversal section (TS) of mastigoneme shafts showing their tubular structure (*d*, arrows). (*e*) Mitochondrion covered with ribosomal subunits. (*f*) Structure of extrusomes at TS (ts), tangential (tas) and longitudinal axial section (as). Scale bars: (*a*) 1 µm, (*b*) 400 nm, (*c*–*f*) 200 nm. Abbreviations: af—adhesive fibres; as—structure of extrusome at longitudinal axial section; b—bacteria; be—belt region; ca—anterior part of corset; cp—posterior part of corset; es—extrusomes; fv—food vacuole; k1—kinetosome of posterior flagellum; k2—kinetosome of anterior flagellum; m—mitochondria; ma—mastigonemes; mah—mastigoneme terminal hairs; mas—mastigoneme shafts; n—nucleus; pf—posterior fibre of corset; pl—r3-associated plate; pr—eukaryotic prey; rh—rhizoplast-like structure; rs—ribosomal subunits; tas—structure of extrusome at tangential section; ft—fibrillar tube associated with belt epiplasm; ts—structure of extrusome at transverse section.

**Figure 7.**
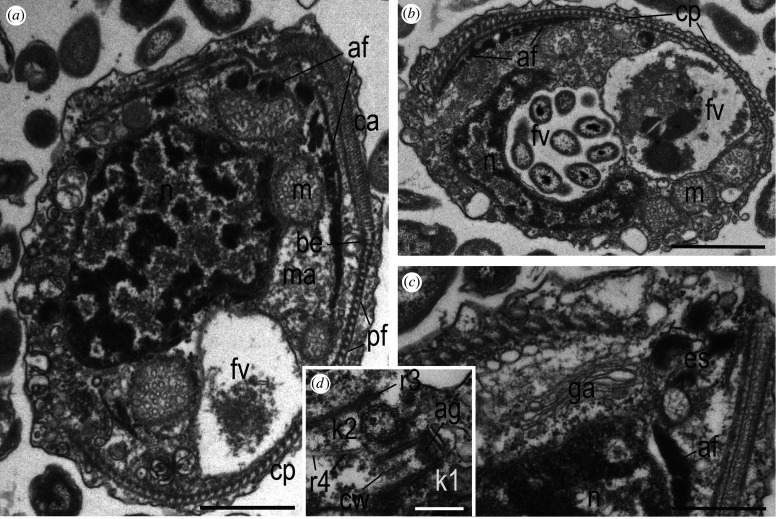
Ultrastructural organization of (*a*–*c*) *Arpakorses versatilis* and (*d*) *Arpakorses idiomastiga*. (*a*) Sagittal section of the cell, ventral side to the left, anterior to the top. (*b*) TS of the cell posterior containing two food vacuoles with bacteria and eukatyotic prey. Dorsal side to the top. (*c*) Golgi apparatus by the nucleus at the middle of the cell. (*d*) orthogonal disposition of kinetosomes characteristic for *A. idiomastiga*. Scale bars: (*a*,*b*) 1 µm; (*c*) 400 nm; (*d*) 200 nm. Abbreviations: af—adhesive fibres; ag—axial granule underneath transversal plate of flagellum; be—belt region; ca—anterior part of corset; cp—posterior part of corset; cw—cart-wheel structure; es—extrusomes; fv—food vacuole; ga—Golgi apparatus; k1—kinetosome of posterior flagellum; k2—kinetosome of anterior flagellum; m—mitochondria; ma—mastigonemes; n—nucleus; pf—posterior fibre of corset; r3 and r4—microtubular roots of kinetosome 2.

The cell body of *A. versatilis* (figures 4*a*,*b* and 5*a*–*d*) is drop-shaped with a rostral outgrowth and cytostome in the apical part of the cell. Cell length is 5.6–8.7 µm, cell width in the widest part is 3.5–6.1 µm. Two acronematic flagella emerge from independent flagellar pockets located subapically in the anterior part of the cell and separated by a protrusion. One flagellum (7.7–15.5 µm) is slightly longer than the other (7.6–11.3 µm). The longer flagellum is equipped with complex tripartite mastigonemes (figure 5*b*,*c*). A prominent food vacuole is located in the posterior part of the cell (figure 4*a*,*b*). In well-fed saturated cells, it can take up to a third of the total volume. The shape of the cells can vary from drop-shaped to elongated teardrop, depending on the food conditions. Cell division is longitudinal (figure 4*b*; electronic supplementary material, video S1). No cysts were detected. The cells can attach to the substrate by one of the flagella, while the second flagellum probably beats to create a water flow near the apical part of the cell. Presumably, these acts are performed to capture bacteria, as evidenced by ultrastructural sections showing bacteria-filled digestive vacuoles (figures 6*a* and 7*b*). We also observed that *A. versatilis* are obligate phagotrophic predators that do not survive in the absence of eukaryotic prey. Cells can make circular movements when attached to the substrate with a flagellum. The engulfment of eukaryotic prey occurs when the prey is in the radius of movement. *Arpakorses* catch prey with the narrow end of the cell and instantly engulf it using a cytostome (electronic supplementary material, video S2 and video S3). Cells can also swim with rotation wide end forward.

#### 3.2.2. *Arpakorses idiomastiga* gen. et sp. nov.

*Arpakorses idiomastiga* (figures 4*c*,*d* and 5*e*–*g*) is characterized by a cell body plan, shape and size similar to *A. versatilis*, but possesses flagella of almost equal length without mastigonemes (figure 5*e*–*g*), and a row of small granules are often seen on the cell surface (figure 4*c*). Cells were never observed to attach to the substrate by the flagella. Movement pattern is a rotation of the entire cell body around the longitudinal axis and a directed movement wide end forward. Well-fed cells acquire a rounded shape and form numerous cell clusters, then settle to the bottom without movement. Cell division is longitudinal (figure 4*d*). Cysts were not detected.

#### 3.2.3. *Telonema papanine* sp. nov.

The cellular shape of *T. papanine* sp. nov. (figures 4*e*,*f* and 5*i*,*l*) varies from oval, ovoid or pear-shaped to almost spherical, depending on the feeding conditions. Longitudinal ribs with a width of 0.07–0.10 µm are visible on the cell surface (figure 5*l*). Cell length is 8.5–13.7 µm, cell width is 4.2–8.3 µm in the widest part. The anterior end of the cell body is elongated, forming a rostrum (figures 4*f* and 5*k*), with a width of 1.3–2.3 µm. Two acronematic flagella of almost equal length (7.4–10.9 µm) extend at the base of the rostrum from independent flagellar pockets separated by a protrusion. Mastigonemes were not observed. Food vacuoles locate posteriorly, and if more than one food vacuole was initially present in the cell, one of them may pass into the daughter cell during division. Division is longitudinal, starting at the front of the cell (electronic supplementary material, video S4). In conditions of lack of eukaryotic prey, bacterial uptake was observed (electronic supplementary material, video S5). Contractile vacuoles of 2.1–3.3 µm in diameter do not have a permanent localization and formed in different parts of the cell (figure 4*f*). Cells actively swim with the wide end forward, rotating around its longitudinal axis, with the flagella directed backwards (electronic supplementary material, video S6). Motionless cells with a thin 2.2–10.7 µm long posterior pseudopodium used for substrate attachment were repeatedly observed in old cultures (figure 4*f*). Immobile cells wrap the flagella around the rostrum.

#### 3.2.4. *Telonema tenere* sp. nov.

The cell body of *T. tenere* sp. nov. (figures 4*g*,*h* and 5*m*,*n*) is oval with a narrow anterior end. Cells of this species are more elongated than other clones and slightly curved inwards. Cell length is 5.8–10.2 µm, cell width is 3.5–7.2 µm. The movement pattern is similar to that of *T. papanine*, but generally smoother. Cells often come into contact with each other during swimming. Several attacks of starving cells on a specimen of its own species were observed (electronic supplementary material, video S7). Such cases clarify the cytoskeleton behaviour during prey engulfment: peripheral corset composed by multilayer structures (see below) is expanding significantly in lateral direction to prepare space for food vacuole formation.

#### 3.2.5. *Telonema rivulare* sp. nov.

Cells of *T. rivulare* sp. nov. (figures 4*i*–*l* and 5*o*) are oval in shape with an elongated rostrum. The rostrum with a diameter of 0.5–1.0 µm is directly cut off at the distal end (figure 4*j*). Length of the cell body is 10–14 µm, width in the widest part is 5.0–9.5 µm. Two short acronematic flagella are approximately of the same length. Flagella are directed backward during the cell swimming. In sedentary cells, the flagella can move freely and often wrap around the body of the cell or directed to its posterior end. Several rows of small granules are often seen on the cell surface (figures 4*k* and 5*o*). The contractile vacuole is located in the posterior half of the cell. Well-fed cells are almost round in shape, with one to three large food vacuoles. Flagellates multiply by longitudinal division on two equal cells (figure 4*l*).

#### 3.2.6. *Telonema subtile* Griessmann, 1913

Morphology of studied representative of previously known species *T. subtile* (clone Tel-1, figure 5*h*) is similar to previous description [17]. Cells are 7.0–9.4 µm long, 3.5–6.1 µm wide, with two 7.5–9.2 µm long isokont flagella equipped with acronemes.

### 3.3. Ultrastructure of the novel telonemid *Arpakorses* spp.

The cell of flagellates *Arpakorses* is covered with a plasmalemma. Telonemid-specific peripheral cytoskeleton (corset) lies just beneath the plasmalemma and consists of microtubules and associated fibrils which form multilayer structures, or multi-layered cytoskeleton, studied in detail by Yabuki *et al.* [17] for *Telonema subtile*. The corset embraces the cell organelles from the dorsal side and from the sides leaving open the anterior and posterior ends of the cell, as well as the ventral side.

**Figure 8.**
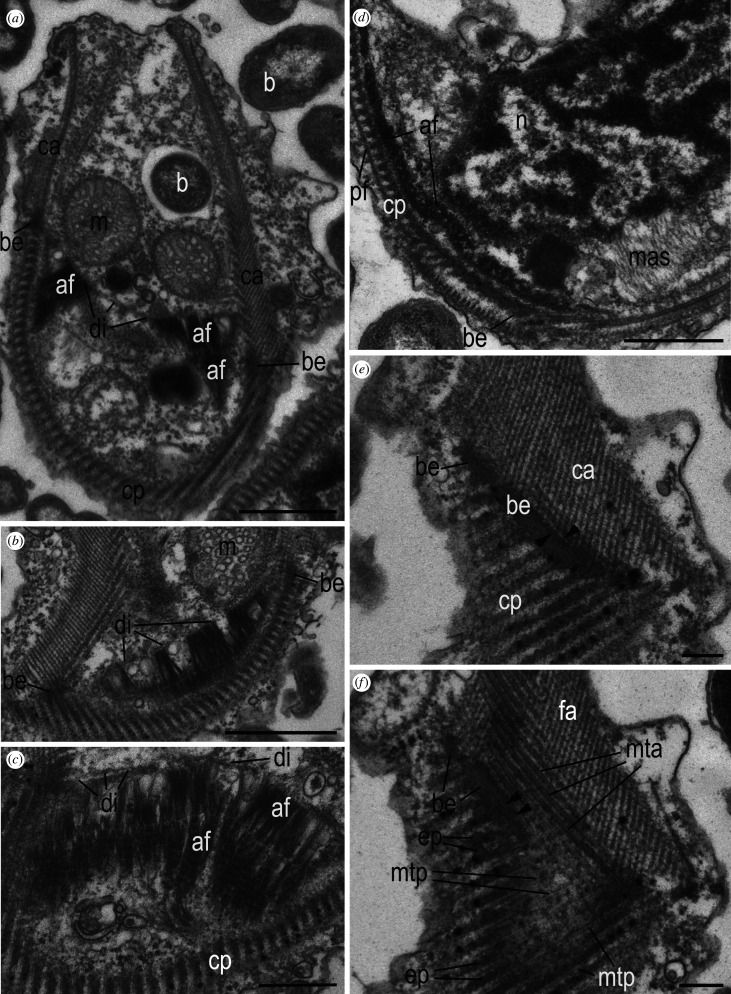
Structure of the belt region and diaphragm of *Arpakorses idiomastiga* (*a*–*c*) and *Arpakorses versatilis* (*d*–*f*). Cell is oriented with anterior end to the top in all figures. (*a*) LS through the cell periphery, (*b*,*c*) belt region and diaphragm associated with AFs, (*d*–*f*) belt in anterior to posterior corset transition, (*e*,*f*) two consecutive tangential sections (60 nm) from surface (*e*) to the cell interior (*f*) direction. Black arrowheads in (*e*) and (*f*) show belt microtubules surrounded by epiplasm, which produce secondary microtubules of posterior part of corset (mtp) with intermediated epiplasmic bands. Scale bars: (*a*,*b*,*d*) 1 µm; (*c*) 500 nm; (*e*,*f*) 250 nm. Abbreviations: af—adhesive fibres; b—bacteria; be—belt region; ca—anterior part of corset; cp—posterior part of corset; di—diaphragm; ep—epiplasm of belt; m—mitochondria; mas—mastigoneme shafts; mta—microtubules of anterior part of the corset; mtp—microtubules of posterior part of the corset; n—nucleus; pf—posterior fibre of corset.

A large nucleus occupies the middle part of the cell (figures 6*a* and 7*a*). Its surface is often invaginated due to tightly fitting mitochondria and digestive vacuoles (figure 6*a*,*e*), which can significantly deform the nucleus (figure 7*b*). Most of the nuclear chromatin is condensed. The less electron-dense euchromatin is labyrinthine and usually does not reach the nuclear membrane (figures 6*a*, 7*a*,*b* and 8*d*). The so-called adhesive fibres (AF; see below) are attached to the nucleus from the outside in some areas, especially from the dorsal side, connecting the surface of the nucleus to the corset (figure 8*d*). The mitochondrion is adjacent to the nucleus and usually underlined by AF (figure 6*e*). A prominent dictyosome of the Golgi apparatus is adjacent to the nucleus on the ventral side (figure 7*c*). The nucleus has a large lateral enlargement of the perinuclear space, which contains tubular mastigonemes (figure 6*b*–*d*). This structure probably constitutes the so-called telonemosome (TS), which has been previously described in *Telonema subtile* (fig. 2D in [17]). Unfortunately, other illustrations of the TS are absent in Yabuki *et al.* [17], so it is difficult to conclude whether it represents an enlargement of the perinuclear space or not. The TS of *Arpakorses* gen. nov. is oval, elongated along the nucleus. Longitudinal sections (LSs) of a pack of mastigonemes show that they have a tubular shaft and a long terminal hair (figures 6*b*–*d* and 8*d*). Tubular flagellar mastigonemes of stramenopiles are also synthesized in the perinuclear space [37–39]. The anterior end of the TS abuts the mitochondria (figure 6*b*). The TS can detach from the nucleus and migrate to the base of the flagellum, where it fuses with the plasmalemma and, as a result of exocytosis, mastigonemes are embedded in the flagellar membrane. The TS of *T. subtile* has a denser matrix compared to *Arpakorses* gen. nov. and also contains a bunch of tubular mastigonems [17], as in most ochrophytes. There are quite a few vesicles of different sizes in the cytoplasm of the ventral part of the cell (figure 7*a*,*b*), apparently representing a storage of membranes for the formation of large vacuoles for food digestion and excretion of undigested residues.

Mitochondria with tubular cristae are rather large and hardly occur outside the corset. The outer membrane of mitochondria is covered with ribosomes (figures 6*e*, 7*a*,*b* and 8*a*), which is extremely rare among protists [40]. Although the ribosome subunits are not as densely located on the mitochondrial surface as in *Arpakorses* gen. nov., this phenomenon has been noted in yeast, and it was shown that such ribosomes are involved in the co-translational transport of proteins into mitochondria [41,42]. The synthesis of proteins occurs directly on the polysomes attached at one end to the outer membrane of the mitochondria. Interestingly, mitochondria are not covered by ribosomes in other studied telonemids [9,10,16,17].

Extrusive organelles (extrusomes) are located just beneath the plasmalemma on the ventral side of the cell. Small groups of extrusomes often can be seen between the corset and the nucleus, where extrusome maturation likely takes place. There is an electron-dense rolled flat ribbon or nested cylinders of different heights (decreasing from the periphery to the centre) inside the extrusome vesicle (figure 6*f*). Discharged extrusomes were not observed; therefore, it cannot be concluded what the longitudinal striation means, coiled ribbon or cylinders. Studied extrusomes are not similar to the extrusive organelles of *T. subtile* [17], which do not possess longitudinal striation and contain an electron-transparent shaft-like structure equal in height to a peripheral electron-dense cylinder having a wheel-like structure made up by 13 components. The *Arpakorses* gen. nov. extrusomes resemble kathablepharid ejectosomes by the presence of longitudinal striation [43,44]. However, in kathablepharids, the central part of ejectosomes is symmetrically depressed at both ends (like an hourglass), and they unfold into ribbons when fired.

#### 3.3.1. Peripheral cytoskeleton (corset) made of microtubules and filaments

The corset consists of two parts, an anterior and a posterior, separated by a spiraling belt made of an electronically dense material and microtubules (figure 8*a*,*b*,*d*–*f*). This structure was named the microtubule organizing boundary (MTOB) by Yabuki *et al.* [17]. The belt consists of 3–5 microtubules immersed in a characteristic electron-dense matrix (epiplasm). The belt starts to the left of the kinetid and goes to the dorsal side of the cell, descending along a wide spiral backward and ends on the right side of the cell, embracing the cell along the periphery. The microtubular bands of the corset run in a spiral from front to back, their number increases and they are arranged in an increasingly shallow spiral (figures 6*b* 8*a* and 9*b*,*g*). Approximately one-third of the anterior end of the cell, the microtubular bands are reinforced by fibrils of various thicknesses perpendicular to them, forming, in accordance with the description of Yabuki *et al.* [17] for *T. subtile*, multi-layered structure (figure 8*a*,*b*,*e*,*f*). Epiplasmic cords appear between the microtubules as they approach the belt. The most posterior microtubules of the anterior part of the corset are actually immersed in the epiplasm and represent a belt (figure 8*d*–*f*). Microtubules of the posterior part of the corset extend from the epiplasm of the belt (which serves as MTOC) perpendicular to it and are, in fact, secondary microtubules (figure 8*e*,*f*). They are covered on the outside with a single-layer posterior fibre (figures 6*b*, 7*a* and 8*d*) as described by Yabuki *et al.* [17] for *T. subtile*. The base of the posterior microtubules is immersed in the belt epiplasm, which accompanies the microtubules for some extent (figure 8*d*–*f*). Wide cross-striated AF extending from the nucleus are attached to the corset (figure 8*a*–*d*) in the area of the transition from the anterior to posterior part of the corset (in the region of the belt). At the other end, the AFs attach to the fibrillar condensation of the cytoplasm, which forms one or two diaphragms along the belt (figure 8*a*–*c*). AF bind the corset with the nucleus, mitochondria and endoplasmic reticulum, and the anterior mitochondrion with the nucleus by microtubules (figures 6*a*,*b*,*e*, 7*a*,*c* and 8*a*–*d*).

**Figure 9.**
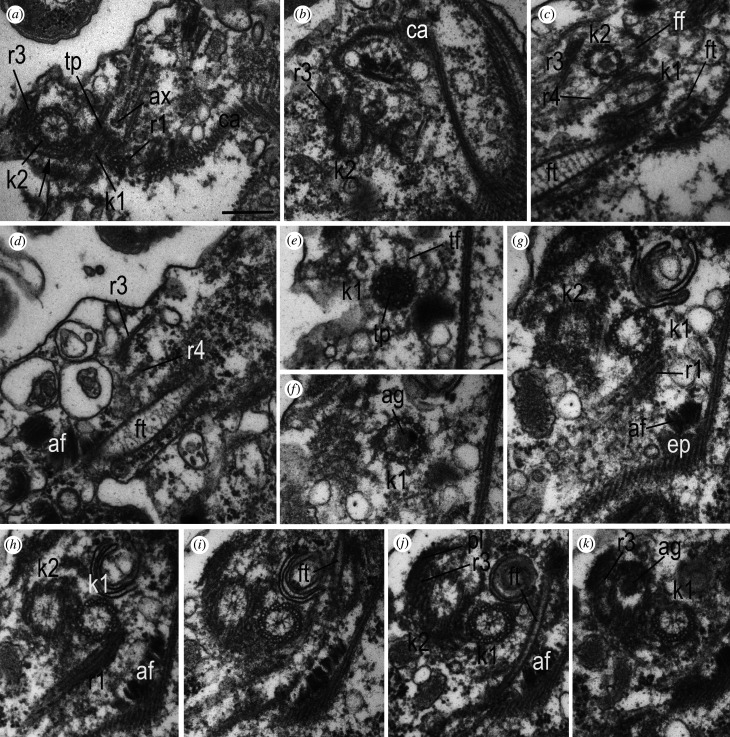
Kinetid structure of *Arpakorses versatilis*. (*a*–*d*) kinetid sections of separate cells, showing the roots and cytoskeletal elements. (*e*–*k*) consecutive serial TSs of kinetosome k1 from distal (*e*) to proximal (*k*) end. Cells oriented dorsal side to the right, anterior end to the top in all sections. Note: triplets of k1 are oriented counter clockwise, thus, our view is from flagellar tip to flagelar base. It means that the k1 gives rise to the left ‘posterior’ flagellum. Arrow on (*a*) shows a fibrillary sheath around posterior surface of k2. Scale bar: 200 nm. Abbreviations: af—adhesive fibres; ag—axial granule underneath transversal plate of flagellum; ax—axoneme; ca—anterior part of corset; ep—epiplasm of belt; ff—fibrillar foot of k2, initiating its microtubular roots r3 and r4; ft—fibrillar tube associated with belt epiplasm; k1—kinetosome of posterior flagellum; k2—kinetosome of anterior flagellum; pl—r3-associated plate; r1—ventral microtubular root of k1; r3 and r4—microtubular roots of kinetosome 2; tf—transition fibres; tp—transversal plate.

**Figure 10.**
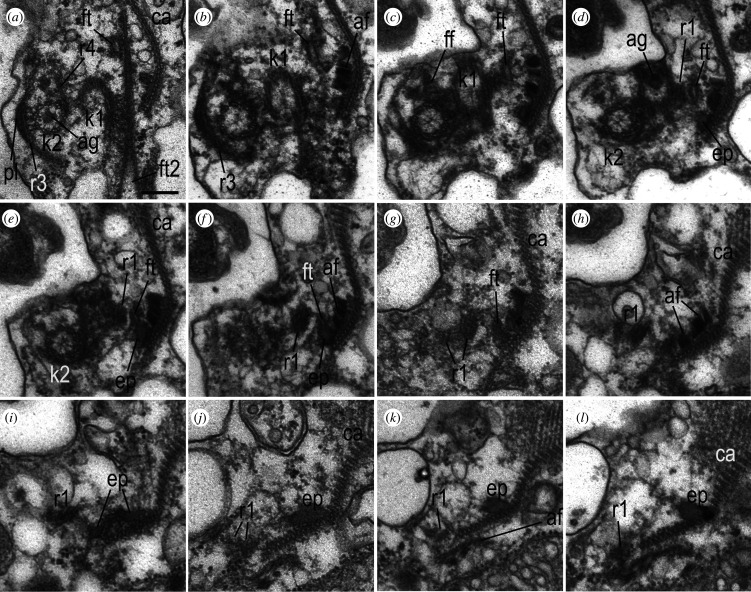
Kinetid structure and belt origin of the *Arpakorses versatilis*. (*a*–*f*) consecutive serial TSs of k2 from distal (*a*) to proximal (*f*) end. (*a*–*l*) consecutive serial sections of belt epiplasm origin (*a*) and its further enlargement (*b*–*l*) from the right to the left side of the cell. Dorsal side of the cell is to the right, anterior end to the top in all sections. Note: triplets of k2 are oriented clockwise; thus, our view is from flagelar base to flagellar tip. It means that the k2 gives rise to the right ‘anterior’ flagellum. Scale bar: 200 nm. Abbreviations: af—adhesive fibres; ag—axial granule underneath transversal plate of flagellum; ca—anterior part of corset; ep—epiplasm of belt; ff—fibrillar foot of k2, initiating its microtubular roots r3 and r4; ft—fibrillar tube associated with belt epiplasm; ft2—another fibrillary tube in kinetid vicinity; k1—kinetosome of posterior flagellum; k2—kinetosome of anterior flagellum; pl—r3-associated plate; r1—ventral microtubular root of k1; r3 and r4—microtubular roots of kinetosome 2.

The process of corset formation during the ontogeny of the telonemid cell has not been studied [10,17]. Here, we show that the posterior part of the cortex is represented by secondary microtubules extending from the belt of its anterior part. The origin of the multilayer structures of the anterior part is not known, but it could be clarified if their connection with kinetid is established. This issue has not been previously discussed in the literature, nor has the possible connections between the structures of the corset and the flagellar roots, which could initiate the growth of microtubules of the anterior part of the corset after cell division, as shown for excavates [45,46]. In *Arpakorses* gen. nov., the belt comes very close to kinetosome 1 and its root R1, but no direct contact with them was found (figures 6*b*, 9*g*–*k* and 10*d*–*l*). If flagellar roots serve as MTOC, then the most likely candidate is the R1, which is the closest to the corset microtubules. It also contains epiplasm around microtubules, as well as a group of 10–12 corset microtubules passing close to it. At the same time, a rather prominent anisodiametric or flattened in some parts fibrillar tube (FT) always passes in the zone of formation of the epiplasm of the belt (figures 9*c*,*d*,*i*,*j* and 10*a*–*g*). Sometimes two FTs pass close to each other (figure 10*a*), with one of them abutting the band of microtubules of the corset, exactly where the epiplasm of the belt is formed (figure 10*c*–*i*). Interestingly, one side of FT is connected with kinetosome 1 (figure 10*a*), or passes in close proximity to R1 (figure 10*c*,*d*). The same FT is also visible in sections of *Lateronema antarctica* (fig. 2 in [9]) and *T. subtile* (electronic supplementary material, figs S3A and S5D in [17]) and its function is unknown.

An epiplasm-bound band of 10–12 microtubules of a corset runs parallel to R1 (figure 10*c*–*l*) and passes in close proximity to its distal end. We assume that the anterior part of the corset can be formed in ontogeny on the basis of kinetid derivatives. For example, it has been shown for Parabasalia, that wide bands of the trichomonads pelta-axostyle complex (in the terminology of Brugerolle [45]) are initiated by the sigmoid fibre extending from kinetosome 2 [39,47]. In jakobids and free-living diplomonads, secondary microtubules of the right and left roots support the ventral side of the cell [46,48]. Investigation of the dividing cells of telonemids is needed to support this statement.

A sleeve with filogranular material, which was noticed by Cavalier-Smith *et al.* [10], is definitely present in *Lateronema antarctica* (fig. 3D in [9]). The function of this structure is unknown.

#### 3.3.2. Adhesive fibres

By the similarity with the retractable rhizoplasts of prasinophytes and centroheliozoans, it can be assumed that AFs of *Arpakorses* can contract and tighten the corset to the nucleus (the cell is stretched) or unclench. This regulates the shape of the corset, and therefore the cell itself. AFs are attached at the distal end to the fibrillar plate of the diaphragm underlying the corset. When AFs contract, they tighten the fibrillar plate and elements of the corset that are attached to the plate, e.g. the mobile microtubular parts of anterior corset to the nucleus, which may be associated with food engulfment, or with the excretion of undigested residues. Besides the broad and short AFs, we found unusual for telonemids thin cross-striated fibre (figure 6*a*) similar to the rhizoplast of green algae [49]. Such rhizoplasts connecting the kinetosomes of prasinophyte algae with the plasma membrane enable the cell to contract [50].

#### 3.3.3. Digestive vacuoles and feeding

Food is engulfed by the cytostome and shifted along the ventral side of the cell. The absence of a cortex here facilitates the formation of large digestive vacuoles. The ventral part of the cell is very labile; many vacuoles, as well as the Golgi apparatus producing lysosomes and vesicles, are localized here. At the same time, cytopharyngeal structures typical for many phagotrophic protists were not visualized. Large digestive vacuoles with an enclosed bodonid prey (figure 6*a*), as well as vacuoles filled with many bacteria (figure 7*b*), are often present in the wide posterior part of the cell. *Arpakorses* can feed on both eukaryotes and bacteria.

Anterior cytostome serves for engulfment of bacteria. Details of the process of eukaryotic prey capture by telonemids are unknown. According to our observations, prey capture occurs on the ventral side of the cell. It is likely that extrusomes are fired and anchor the prey cell upon contact with the plasmalemma. Then, a large invagination is formed as a result of the divergence of the lateral parts of the corset (corset opening). This process can be imagined as a ‘retraction of the abdomen’ for clarity. The predator becomes flattened dorsoventrally, as seen in figure 7*b*. This is probably achieved by the contraction of the AFs between the corset and the diaphragm. The adherent prey cell is then drawn into the food invagination of a predator, which closes to form a food vacuole. Further, the food vacuole migrates to the posterior end of the cell, where it fuses with lysosomes and the prey is digested.

#### 3.3.4. Kinetid structure

The kinetid is located ventrally at the anterior end of the cell (figure 6*b*). Kinetosomes lie in different planes at an angle of 90–170° to each other. In *A. versatilis* gen. et sp. nov., the kinetosomes are more often orthogonal (figure 7*d*), while in *A. idiomastiga* gen. et sp. nov. they lie at an obtuse angle of up to 180°, or antiparallel (figure 9*h*–*k*), in which case one kinetosome is located closer to the cell surface (ventral), the other is deeper under it (dorsal).

To define the anterior and posterior flagella in telonemids, we compared our studies with published data. Yabuki *et al.* [17] have identified a single mastigonemе on the flagellum originated from ventral kinetosome and determined the numbering of flagella in *T. subtile* by analogy with stramenopiles, in which the anterior flagellum is covered with tubular mastigonemes, and the posterior one is smooth. Tubular mastigonemes are present on one of the flagella in *Lateronema antarctica* [9,10] and *T. subtile* [16,17], but they exfoliate from the flagellum at chemical fixation and whole-mount preparations. Even in those rare cases when they are retained, it is impossible to determine which flagellum is anterior and which is posterior by whole-mount preparation. It was not possible to find mastigonemes on TEM sections, and those shown in one *T. subtile* cell only on two sections (figure 2*k*; electronic supplementary material, fig. S1E,F in [17]) correspond to simple hairs in thickness, not tubular mastigonemes, which is consistent with the opinion of other authors [10]. Apparently, tubular mastigonemes detach from the flagellum during fixation (fig. 10 in [10]), while simple hairs are partially preserved, but they are characteristic only of the posterior flagellum of telonemids. In fig. 12A of *Lateronema* in Cavalier-Smith *et al.* [10], simple hairs are located just on the posterior short flagellum, which usually adjoins the cell body and originates from dorsal kinetosome. It is likely that confusion occurred in the study of Yabuki *et al.* [17], who identified the anterior flagellum from a dubious mastigoneme; the anterior flagellum should be flagellum B in our opinion. It follows that in telonemids, the kinetosome of the anterior flagellum is ventral, while the kinetosome of the posterior flagellum is dorsal. This also determines the numbering of kinetosomes: as in all stramenopiles, the flagellum with tubular mastigonemes originates from kinetosome 2 (which is the younger centriole), and the smooth (or bearing simple hairs) flagellum originates from kinetosome 1 (the older mother centriole) [51]. The correct kinetosome numbering is essential for elucidating the homology of the roots extending from the kinetosomes. In the present work, we determined that in *Arpakorses* gen. nov., the ventral kinetosome is kinetosome 2 (k2) with extending anterior flagellum bearing tubular mastigonemes, and the dorsal kinetosome is kinetosome 1 (k1) with a posterior flagellum.

Judging by the orientation of microtubular triplets in kinetosomes in cross-sections (figure 9*h*,*i*), k2 is directed to the right and k1 is directed to the left. Both kinetosomes have a similar structure: the proximal part contains a cart-wheel structure, which occupies about two-thirds of the kinetosome, the distal part holds a large axial granule and the transversal plate is situated above (figures 9*e*–*k* and 10*a*–*d*). Two central axonemal microtubules extend from an axosome located above the transversal plate (figure 9*a*). The axial granule in the flagellar transitional zone is unknown in protists [52], but was recently described in the flagella of choanocytes [53] and epithelial cells of sponge larvae [54,55].

#### 3.3.5. Flagella root structure and homology with stramenopiles, alveolates and rhizarians

Two microtubular roots extend from the distal end of the fibrillar foot located on the anterior surface of k2 (figure 9*a*–*d*), and pass back along the ventral and dorsal sides of k2. A prominent ventral root (R3) consists of eight microtubules and associated at the base with the ventral fibrillar plate (figures 6*b*, 9*j* and 10*a*,*b*). R3 runs under the plasmalemma to the right and slightly backward, terminating at the right edge of the corset, approximately in the middle of the cell (figure 9*d*). Inconspicuous R4 of 1–2 microtubules runs from the foot along dorsal side of k2 and continues parallel to R3 (figures 9*c,d* and 10*a*).

A bundle of 5 (2 + 3) microtubules (R1), immersed in an electron-dense matrix, originates from k1 on its dorsal side (figure 9*a*,*g*,*h*). This root passes to the left towards the ventral surface of the cell. R2 was not found in the kinetid of *Arpakorses* gen. nov.

The structure of the kinetid in telonemids has not been studied before. Only a few details can be seen in the figures by different authors. For example, R1 is visible near kinetosome A (k1 in present paper) of *T. subtile* (electronic supplementary material, fig. S3D in [17]).

In most stramenopiles, two microtubular roots originate from each kinetosome [51,56]. Despite the fact that in some protists some roots may be reduced, such a set is considered to be the most conservative and closest to the ancestral state of stramenopiles, and probably all rhizarians and alveolates [56].

It can be argued that in *Arpakorses* gen. nov. k2 of the anterior flagellum has microtubular roots R3 and R4, while k1 of the posterior flagellum produces R1 microtubular root only.

The flagellar apparatus structure of protists has been reviewed by Yubuki & Leander [56]. These authors have unambiguously shown that each supergroup of eukaryotes is characterized by peculiar kinetid structure, and the flagellar roots in particular. *Arpakorses* gen. nov. kinetid possess some elements especially similar to rhizarians: two so-called ventral posterior microtubular roots originating from k2 and passing parallel to each other in *Paracercomonas*, *Cercomonas* and probably in *Heteromita* [57,58]*.* A cercozoan *Katabia* has a fibrillar plate at the base of the posterior microtubular band originating from the basal body of anterior flagellum [59].

### 3.4. Evolutionary and ecological significance of telonemids

Telonemia has been proposed to form the sister group to the large eukaryotic supergroup SAR [7]. This phylogenetic position places strong emphasis on the telonemids to infer the morphology and behaviour of the common ancestor of stramenopiles, alveolates and rhizarians. Until now, however, the morphology and cellular features of telonemids have only been observed in two species [9,17]. Here, we provide detailed morphological and ultrastructural observations for six new species, including a new genus. They are quite similar in external morphology (figure 4) but can be distinguished even with light microscopy (table 1). Characterizing the cellular biology of diverse telonemid species is important to infer ancestral states, because in spite of external similarities and the presence of tubular mitochondrial cristae uniting them with all SAR lineages, we show that there are significant differences at the ultrastructural level between the described representatives of the three known genera *Telonema*, *Lateronema* and *Arpakorses* gen. nov. Interestingly, these genera individually bear ancestral characters similar to different SAR taxa.

**Table 1.** Comparison of morphological characteristics and habitats of telonemids.

species, clone	cell length (µm)	cell width (µm)	cell surface	length of flagella (µm)	tripartite tubular mastigonemes	flagella wrap around the cell	equal length of flagella	habitat	peculiar features
*Arpakorses versatilis*, clone P-1	5.6–8.7	3.5–6.1	—	7.7–15.5; 7.6–11.3	yes	no	no	marine	cells attached to the substrate with one flagellum
*A. idiomastiga*, clone P-2	5.6–8.7	3.5–6.1	granules	3.3–7.2	no	no	yes	marine	form numerous cell clusters, creates a flow of water with a free flagellum
*Telonema papanine*, clone Tel-2	8.5–13.7	4.2–8.3	ribs	7.4–10.9	no	yes	yes	freshwater	cells attached to the substrate by pseudopodia
*T. tenere*, clone Tel-3	5.8–10.2	3.5–7.2	—	3.5–8.5	no	no	yes	marine	cell body more elongated and slightly curved inwards; cells often come into contact with each other during swimming; cannibalism observed
*T. rivulare*, clone Tel-4	10–14	5.0–9.5	granules	4.0–8.5	no	yes	yes	freshwater	shorter flagella relative to cell body length
*T. subtile*, clone Tel-1	7.0–9.4	3.5–6.1	—	7.5–9.2	no	no	yes	marine	cells swim with sharp jerks and alternately change motion from rectilinear to rotation; length of flagella usually equal to the cell body length
*Lateronema antarctica* [9]	8–16	6–12	vesicles and rods	significantly longer than the cell length	yes	not mentioned	no	marine	one flagellum points sideways during fast swimming; cells may suddenly change direction during swimming

The main trait uniting stramenopiles is the presence of complex tripartite mastigonemes on the anterior flagellum [60]. They are composed of a hollow base and tubular shaft, as well as non-tubular distal fibres [61]. Although absent in *Telonema*, tripartite mastigonemes have been described in *Lateronema* [9,10] and we now show that they are also present in *Arpakorses versalitis* gen. et sp. nov. Given the complex morphology and ontology of tripartite mastigonemes, it has been previously suggested that these flagellar hairs have a common origin [16]. Assuming their homologous origin, it is thus plausible that an ancestor of stramenopiles and telonemids evolved tripartite mastigonemes, which were then lost in alveolates and rhizarians. Following their common origin, tripartite mastigonemes may have evolved different functions. We infer that the long complex mastigonemes in *Arpakorses* gen. nov. increase the flow of water near the apical part of the cell for capturing prey, and obviously make the cell propulsion by its posterior flagellum more efficient during swimming*.* By contrast, stramenopile mastigonemes were proposed to have a swimming-associated function, pulling the cells forward [62]. This raises again a question of tubular mastigonemes function among TSAR representatives.

One of the main characters defining the alveolates is a layer of flattened vesicles, named cortical alveoli, underlying the plasma membrane [63]. The alveoli are known under the cell wall of glaucophytes and have been found also in haptophytes. Discussing these facts, Takahashi *et al*. [64] supposed the presence of alveolate pellicle in the common ancestor of Glaucophyta, Alveolata and Haptophyta (i.e. in the common ancestor of eukaryotic supergroups Archaeplastida, TSAR and Haptista). Alveoli were not found in the new species described here but similar structures were reported in *Lateronema* in the form of large flattened vesicles containing dense material just beneath the cell membrane [9]. These peripheral vacuoles in *Lateronema* and the cortical alveoli of Alveolates could be homologous, evolving as an adaptation for strengthening the cell [65]. In addition to the alveoli, another structure in telonemids resembles some members of alveolates. Indeed, it was shown that the anterior microtubules of *Telonema subtile* form a rolled-up sheet which superficially resembles the specialized penetrating/feeding apparatus (apical complex) of apicomplexans and predatory colpodellids [17,66]. However, the telonemids studied here ingest food subapically or ventrally but not apically.

The diagnostic character of Rhizaria is the presence of fine pseudopodia [60], which were revealed in *Telonema papanine* sp. nov*.* Extrusive organelles of *Telonema subtile* and cercozoan *Heliomorpha* are also extremely similar [17,67]. Yabuki *et al.* [17] pointed out that the structure of extrusomes of *T. subtile* in transverse section is similar to those of several monadofilosan cercozoans, but distinguished by a longitudinal element. However, extrusomes in *Arpakorses* gen. nov. are different and more similar to kathablepharid ejectosomes. In that respect, kinetid structure of *Arpakorses* gen. nov. is similar to that of rhizarians (see above).

All telonemid genera possess a very complex cytoskeleton that is not found in other eukaryotes. Cavalier-Smith *et al.* [10] argue that two major parts of the row of anterior microtubules in *T. subtile* described by Yabuki *et al.* [17] resemble the two posterior centriolar roots of excavate protists (i.e. the A2 layer is like the I-fibre-associated right root and the P layer is like the left root with a C fibre [46]). The presence of a highly intricate multi-layered cytoskeleton may indicate that telonemids have retained characteristics of an ancestral complex organization of corset before the cytoskeleton was differentially reduced in other eukaryotic groups [7]. It seems that adhesive fibrils can change the shape of the corset in telonemids. Similar retractable rhizoplast-like structures were found in prasinophytes, centrohelids and other protists.

Both microscopic observations and results of environmental sequencing indicate that telonemids are widely distributed [11,12,15]. They can be a dominating component in protozoan communities on certain occasions [9], and are regularly observed in marine and brackish waters [12–14,30]. Telonemia probably play a significant ecological role as an important consumer of bacteria and microeukaryotes. The versatility of their feeding should be considered when reconstructing the microbial networks in both marine and freshwater habitats. The discovery and cultivation of *T. rivulare* and *T. papanine* clearly indicate the existence of freshwater telonemids. The presence of freshwater-dwelling environmental sequences in different deeply diverging clades of Telonemia and their complex distribution across the tree argue for independent and multiple transitions from seawater to freshwater. Ancestral telonemids were probably marine and cold-water.

In conclusion, telonemids are morphologically very complex, combining traits of different SAR lineages and several other groups of protists. The strikingly different ultrastructure of different species suggests that telonemids underwent a complex evolutionary pathway. Considering that there are likely to be dozens of telonemid genera, further research and taxon sampling is needed before we draw strong conclusions. Future directions could include sequencing the full-length ribosomal DNA (as in [68]) from clonal cultures and environmental samples in order to obtain a strong phylogenetic framework of telonemids. This phylogenetic framework could then be used to understand the distribution of key cellular characteristics within telonemids to infer the morphology of the common ancestor of Telonemia and SAR. Additionally, it will be vital to establish more clonal cultures of morphologically uncharacterized environmental lineages of telonemids. Microscopic investigations of such novel representatives of Telonemia would provide better understanding of the origin and the evolution of main synapomorphies and cellular innovations of the SAR supergroup. Since telonemids depend on eukaryotic prey, it is difficult to isolate and study them, as they cannot be easily extracted from their ecological network. For this reason, they are overlooked and understudied in microbial communities, where they probably occupy the upper levels of microbial trophic networks and probably play crucial roles in the flow of energy and nutrients. This is an important gap in our understanding of microbial diversity and food webs that should be addressed in future research.

## 4. Taxonomic summary

Taxonomy: Eukaryota; Diaphoretickes; TSAR; Telonemia Shalchian-Tabrizi 2006.

*Arpakorses* n. gen. Tikhonenkov and Karpov.

Diagnosis: Phagotrophic biflagellate drop-shaped protists with a rostral outgrowth and cytostome in the apical part of the cell. Acronematic flagella emerge subapically from independent flagellar pockets. Distal part of kinetosomes holds a large axial granule under transversal plate. Peripheral cytoskeleton embraces the cell organelles from the dorsal side and from the sides and consists of two parts, separated by a spiraling belt made of an electronically dense material and microtubules. AFs connect the surface of the nucleus to the peripheral cytoskeleton. Most chromatin of the interphase nucleus is permanently condensed. The outer membrane of mitochondria is covered with ribosomes. Extrusive organelles represented by electron-dense rolled flat ribbon or nested cylinders of different heights inside the vesicle.

Etymology: from Greek *αρπάκτικο* (predatory) and *κορσ*ές (corset).

Zoobank registration. urn:lsid:zoobank.org:act:8F561E84–4C63-431D-9866-F7DA312AE751.

Type species: *Arpakorses versatilis.*

*Arpakorses versatilis* n. sp. Borodina, Karpov, Zagumyonnyi, Belyaev, Prokina and Tikhonenkov.

Diagnosis: Cell length is 5.6–8.7 µm, cell width in the widest part is 3.5–6.1 µm*.* Kinetosomes are orthogonal. One flagellum is slightly longer than the other and equipped with complex tripartite tubular mastigonemes. Mastigonemes synthesized in the lateral enlargement of the perinuclear space of the nucleus. The cells attach to the substrate by one of the flagella and make circular movements. Longitudinal cell division.

Type figure: figure 4*a* illustrates a live cell of strain P-1.

Gene sequence: The SSU rRNA gene sequence has the GenBank accession number OM415987.

Type locality: water column of the Kara Sea.

Etymology: species name means ‘rotating, swivel, non-constant’ (Lat.).

Zoobank registration: urn:lsid:zoobank.org:act:E965389F-662A-4ED1-915B-7AA0520651F0.

*Arpakorses idiomastiga* n. sp. Borodina, Karpov, Zagumyonnyi, Belyaev, Prokina and Tikhonenkov.

Diagnosis: Cell length is 5.2–8.5 µm, cell width in the widest part is 2.9–5.8 µm*.* Flagella of almost equal length without tripartite mastigonemes. Cells do not attach to the substrate by flagella and swim rotating the cell body around the longitudinal axis wide end forward. Well-fed cells form numerous cell clusters. Longitudinal cell division.

Type figure: figure 4*c* illustrates a live cell of strain P-2.

Gene sequence: The SSU rRNA gene sequence has the GenBank accession number OM415986.

Type locality: water column of the Kara Sea

Etymology: species name means ‘bearing flagella of the same length’ (Greek).

Zoobank registration: urn:lsid:zoobank.org:act:E3A3F46A-A6DE-4A97-9FE5-4B397472E2AA.

*Telonema papanine* n. sp. Zagumyonnyi and Tikhonenkov.

Diagnosis: Cell ovoid, pear-shaped or almost spherical with rostrum and longitudinal ribs on the surface. Cell length is 8.5–13.7 µm, cell width is 4.2–8.3 µm in the widest part*.* Two acronematic flagella of almost equal length without mastigonemes. Contractile vacuoles formed in different parts of the cell. Motionless cells form long posterior pseudopodium and can wrap the flagella around the rostrum.

Type figure: figure 4*e* illustrates a live cell of strain Tel-2.

Gene sequence: The SSU rRNA gene sequence has the GenBank accession number OM480747.

Type locality: wet moss and sand on Champ Island, Franz Josef Land.

Etymology: named after Dr Ivan Dmitrievich Papanin, Arctic explorer, head of the first drifting ice station, North Pole-1.

Zoobank registration: urn:lsid:zoobank.org:act:D97BEF50-4A7B-4A04-9A13-FD4DF0BDA51F.

*Telonema tenere* n. sp. Belyaev, Borodina and Tikhonenkov.

Diagnosis: Cell body is elongated oval with narrow anterior end and slightly curved inwards. Cell length is 5.8–10.2 µm, cell width is 3.5–7.2 µm*.* Cells often contact with each other during swimming.

Type figure: figure 4*g* illustrates a live cell of strain Tel-3.

Gene sequence: The SSU rRNA gene sequence has the GenBank accession number OM415985.

Type locality: sand of the marine littoral zone of the White Sea.

Etymology: species name means ‘tender’ (Lat.), cells are in frequent contact with each other.

Zoobank registration: urn:lsid:zoobank.org:act:F752DDF8-3C33-434A-AFFB-85FCA04D80A3.

*Telonema rivulare* n. sp. Prokina and Tikhonenkov.

Diagnosis: Cell is oval in shape with an elongated rostrum, which is directly cut off at the distal end. Length of the cell body is 10–14 µm, width in the widest part is 5.0–9.5 µm. Two acronematic flagella of approximately the same length. Flagella can wrap around the body in sedentary cells. Several rows of small granules are on the cell surface. Contractile vacuole located posteriorly. Cell division is longitudinal.

Type figure: figure 4*i* illustrates a live cell of strain Tel-4.

Gene sequence: The SSU rRNA gene sequence has the GenBank accession number OM403114.

Type locality: bottom detritus with sand of Sakhray River, Adygea, Russia.

Etymology: species name means ‘riverine’ (Lat.).

Zoobank registration: urn:lsid:zoobank.org:act:43531A0D-0EC5-490B-A5D0-6697298A5847.

## Data Availability

All data are available in the main text or the electronic supplementary material [69]. DNA sequences: GenBank accession numbers OM388565, OM403114, OM415985–OM415987, OM480747.
